# Common genetic susceptibility to DCIS and invasive ductal carcinoma

**DOI:** 10.1186/s13058-016-0719-z

**Published:** 2016-06-10

**Authors:** Victoria Sopik, Steven A. Narod

**Affiliations:** Women’s College Research Institute, 76 Grenville Street, Toronto, Ontario Canada

In a recent issue of *Breast Cancer Research*, Petridis and colleagues report a failure to detect any genetic polymorphism which predisposes to ductal carcinoma in situ (DCIS) but not to invasive ductal carcinoma (IDC) or vice versa [1]. They genotyped 5067 cases of DCIS, 24,584 cases of IDC, and 37,467 non-cancer controls for 76 different single nucleotide polymorphisms (SNPs), each of which has been shown to be associated with breast cancer susceptibility in previous studies [2, 3]. They found no significant difference in the magnitude of the associations for DCIS and IDC for any of the 76 loci. From this, the authors conclude that the genes responsible for DCIS and IDC are, by and large, the same and that larger studies are required to determine if susceptibility loci specific to DCIS exist.

The authors base the rationale for this study on the premise that DCIS and IDC are distinct pathologies, in that DCIS is non-invasive and is a non-obligate ‘precursor’ lesion. Accordingly, they seek a biological basis which helps underpin the distinction. A simpler and parsimonious explanation for the overlap in gene sets is that DCIS and IDC represent various phases of the same disease process, rather than distinct forms of cancer, as we have recently proposed [4]. In this sense, it is unlikely that they will identify genes which predispose to stage 0 but not to stage I breast cancer (as one would not expect to find genes that predispose to stage II but not stage I cancer). If one wishes to study the possibility that host factors are associated with cancer stage at presentation—and with rate of progression—it is better to study breast cancers in their entire range (from stage 0 to stage IV) and to look for overall trends in prevalence.
