# Supplementary material for: Common genetic susceptibility to DCIS and invasive ductal carcinoma
Source: Breast Cancer Res. 2016 Jun 10;18:60. doi: 10.1186/s13058-016-0719-z (PMC4902925; doi:10.1186/s13058-016-0719-z)
Supplement: Related texts

# Reply: Authors’ response

**Authors:** Christos Petridis, Sarah E. Pinder, Ian Tomlinson, Rebecca Roylance, Elinor J. Sawyer

In response to the comments by Sopik and Narod we would like to point out that the premise of our study was not that DCIS and IDC are distinct pathologies and we were not therefore seeking a biological basis to explain this distinction. Indeed we state that: “45–78 % of all invasive breast cancers are associated with DCIS and it is hypothesized in the majority of these cases that the invasive component has arisen from the DCIS as they generally share the same somatic genetic changes” [1]. We agree with the obvious explanation, which is that DCIS and IDC represent various phases of the same disease process.

Nevertheless, it is also clear that not all DCIS progresses to an invasive lesion. In particular, there are concerns regarding over-diagnosis and over-treatment of DCIS through screening programmes [5]. Current methods for accurately predicting the behaviour of an individual DCIS lesion are poor, with many researchers attempting to identify molecular biomarkers that can be used to distinguish between aggressive and non-aggressive DCIS with, to date, little success. The rationale for our study was to ascertain whether a subgroup of non-aggressive DCIS could be identified by examining low-risk genetic susceptibility loci, rather than looking at somatic changes within DCIS.

In our study we examined the extent to which DCIS without associated invasive disease (5067 cases) and IDC (24,584 cases) share low-risk susceptibility loci and whether there were any differences in the strength of the associations. We found no differences, although there were some DCIS-specific loci that did not reach genome-wide significance. Therefore, our data largely support the theory that DCIS and IDC are, in fact, a continuum of the same disease, but we cannot exclude the possibility that there may be some low-risk susceptibility loci that have a strong association with stage 0 and a weaker association with stage I breast cancer. Identifying any such loci is important as it would identify a subset of DCIS that has a low risk of progression.

## Abbreviations

DCIS, ductal carcinoma in situ; IDC, invasive ductal carcinoma; SNP, single nucleotide polymorphism
